# The current state of inflammation-related research in prostate cancer: a bibliometric analysis and systematic review

**DOI:** 10.3389/fonc.2024.1432857

**Published:** 2024-09-17

**Authors:** Weida Li, Jian Wang

**Affiliations:** Affiliated Hospital of Guangdong Medical University, Zhanjiang, China

**Keywords:** prostate cancer, inflammation, bibliometrics, visual analytics, inflammation-related markers

## Abstract

**Background:**

Prostate cancer (PCa) is the second most prevalent malignancy among men globally. The diagnosis, treatment, and prognosis of prostate cancer frequently fall short of expectations. In recent years, the connection between inflammation and prostate cancer has attracted considerable attention. However, there is a lack of bibliometric studies analyzing the research on inflammation within the domain of prostate cancer.

**Research methods:**

We utilized the Web of Science Core Collection (WOSCC) as our data source to extract articles and reviews related to inflammation in prostate cancer, published up until April 12, 2024. The collected data underwent meticulous manual screening, followed by bibliometric analysis and visualization using the Biblioshiny package in R.

**Results:**

This study encompasses an analysis of 2,786 papers focusing on inflammation-related research within the realm of prostate cancer. Recent years have seen a significant proliferation of publications in this area, with the United States and China being the foremost contributors. The most prolific author in this domain is Demarzoam, with Johns Hopkins University standing out as the most influential institution. The leading journal in disseminating these studies is PROSTATE. Keyword co-occurrence analysis reveals that ‘inflammation-related biomarkers’, ‘inflammation index’, and ‘tumor immune microenvironment’ represent the current research hotspots and frontiers.

**Conclusion:**

The findings of this bibliometric study serve to illuminate the current landscape of inflammation-related research in the field of prostate cancer, while further augmenting the discourse on inflammation-mediated cancer therapeutics. Of particular note is the potential of these discoveries to facilitate a more nuanced understanding among researchers regarding the interplay between inflammation and prostate cancer.

## 1 Introduction

Prostate cancer is a prevalent malignancy among men and ranks as the fifth leading cause of cancer-related mortality in this demographic (1). In 2020, the global incidence of new cases was approximately 1.4 million, with fatalities nearing 375,000. It remains the most common cancer in men across more than half of all countries, with incidence rates varying significantly from region to region—ranging from 6.3 to 83.4 per 100,000 (2). Regions with the highest incidence include Northern and Western Europe, the Caribbean, Australia/New Zealand, North America, and South Africa (3). A substantial relationship exists between inflammation and the onset of prostate cancer. Inflammation is the body’s protective response to injury, infection, or other stimuli, typically characterized by redness, pain, and tissue dysfunction. Although the development of prostate cancer is associated with multiple genetic factors, these genes have been linked to inflammatory metabolic pathways. For instance, the MIC 1 gene, part of the transforming growth factor β (TGF-β) superfamily, acts as a macrophage inhibitory cytokine (4). Studies, such as those by Comito G and colleagues, have identified an association between the overexpression of M2 macrophages in prostate tumors and factors such as extracapsular extension and early biochemical recurrence (5). In Swedish research on prostate cancer, a significant number of patients exhibit nonsynonymous variations in the gene H6D (6). Concurrently, extensive modifications in the IL 1 RN gene—related to the interleukin-1 family and inhibiting pro-inflammatory IL 1 α and IL 1 β—were observed among many patients (7). Furthermore, in the immune response, T cells, especially cytotoxic T lymphocytes (CTLs), are critical in the immune-mediated clearance of tumors, while tumor-infiltrating lymphocytes (TILs) are vital for sustaining the anti-tumor immune response (8). A meta-analysis by Linghao Meng et al. incorporating data from 12 studies and 8,083 patients, has shown that the systemic immune-inflammation index (SII) is an effective marker for assessing the global state of immune inflammation. The SII has demonstrated diagnostic and prognostic value in various urological cancers, including prostate, renal, bladder, and upper urinary tract cancers, highlighting the close correlation between inflammation and the emergence, progression, and prognosis of prostate cancer (9). Additionally, persistent chronic inflammation may increase prostate cancer risk, as high levels of inflammatory cell infiltration, such as lymphocytes and macrophages in prostate tissues, can produce cytokines and chemicals leading to DNA damage and inflammation-related cellular changes, thereby promoting tumor development. Moreover, other microbes, including bacteria and viruses that stimulate prostate inflammation, may further enhance the development of prostate cancer (10, 11). Notably, Propionibacterium acnes, a pro-inflammatory bacterium linked to skin conditions such as acne and other inflammatory states including endocarditis and postoperative infections, has been implicated in prostate inflammation and cancer since its initial association in 2005. These pathogens can induce chronic inflammation and abnormal cell proliferation, thus elevating the risk of prostate cancer (12).

In the realm of prostate cancer, amidst a wealth of burgeoning research and rapid advancements concerning inflammation, there exists a compelling imperative to dedicate substantial time and intellectual vigor towards the curation and scrutiny of publications, thereby acquiring requisite and pertinent insights. Bibliometrics emerges as a venerable methodology within the domain of systematic evaluation studies, elucidating the contributions of various nations and institutions, identifying stalwart researchers and potential collaborators, and delineating the evolutionary trajectories of research paradigms (13). Notably absent, however, are bibliometric analyses pertaining to prostate cancer and inflammation. To address this lacuna in scholarly inquiry, we proffer a meticulously crafted exposition employing bibliometric methodologies, affording an accurate depiction of the centrality of inflammation-related investigations within the sphere of prostate cancer research.

## 2 Methods

### 2.1 Data source

The Web of Science (WOS) database serves as the primary data source for this investigation. Within the academic sphere, WOS is considered one of the most comprehensive bibliographic databases, extensively utilized in bibliometric studies. Accordingly, we selected the Web of Science Core Collection (WOSCC) to facilitate our search for publications pertaining to inflammation and prostate cancer. (The data of this study was searched from the WOSCC; thus, no ethical approval is required.)

### 2.2 Data search strategy

On April 13, 2024, a thorough literature retrieval was conducted within the WOSCC for articles and reviews related to inflammation in prostate cancer published up to April 12, 2024. The search strategy employed was: TS = (“inflammation”) AND (“prostate cancer” OR “prostatic cancer” OR “prostate neoplasm*” OR “prostatic neoplasm*”). Our dataset exclusively includes papers published in English, focusing solely on articles and reviews, and excluding other forms of publication such as letters and conference proceedings. Subsequently, titles, keywords, abstracts, and even full texts were meticulously reviewed to exclude literature irrelevant to the research theme.

### 2.3 Data extraction and collection

**Figure 1:**
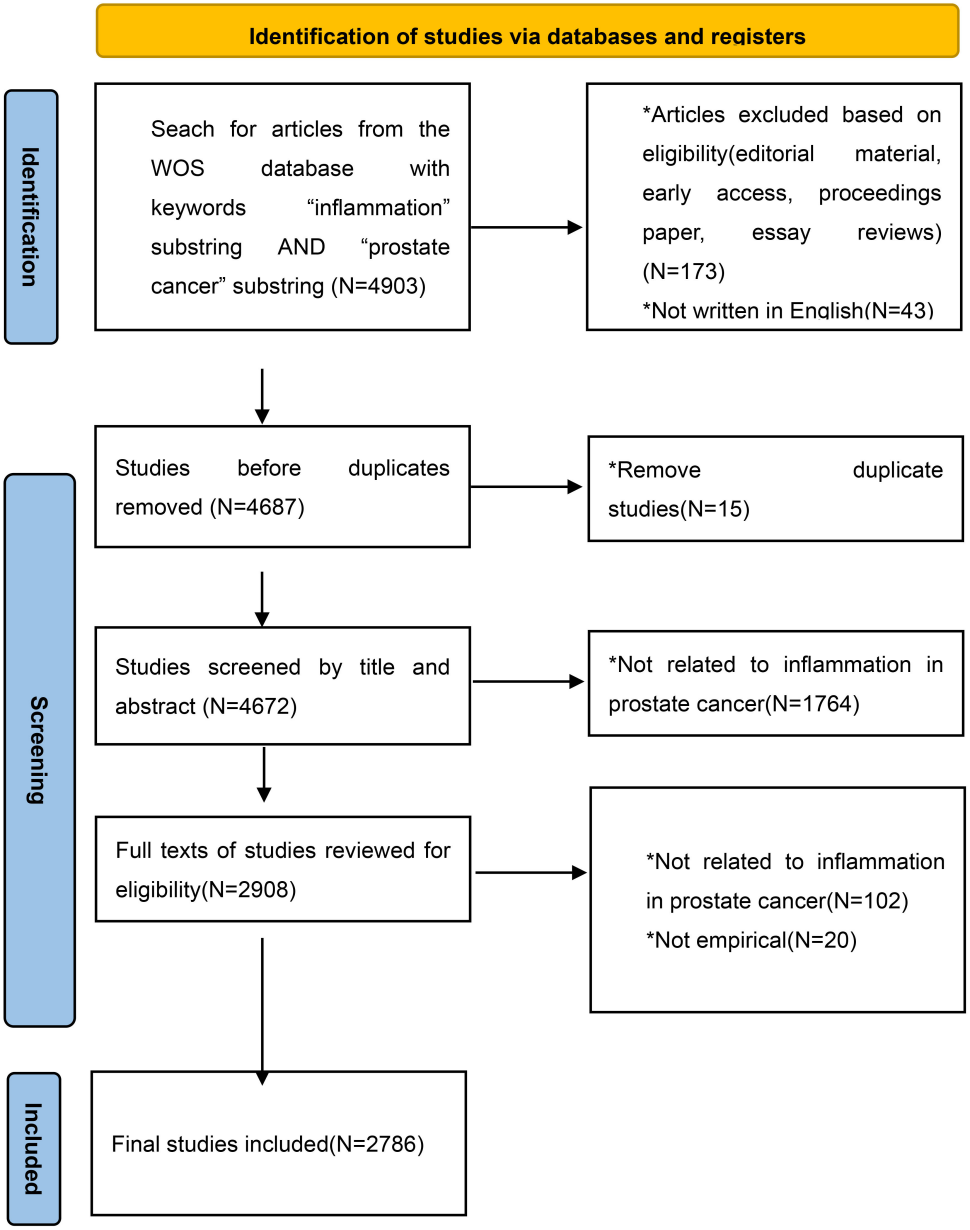
Data collection and filtering flow diagram.

A total of 1,409 documents, encompassing complete records (authors, source, title, publication, abstract, keywords, addresses, references, grants, etc.), were retrieved from the WOS database and exported in plain text format to the Bibliometrix R-Tool (http://www.bibliometrix.org), an R package developed by Aria and Cuccurullo (14), which offers a comprehensive toolkit for quantitative bibliometric and scientometric research. Concurrently, basic analyses and plotting were performed using Microsoft Excel 2022, as detailed in **Figure 1**.

### 2.4 Data analysis and visualization

The analysis of the data was twofold. Initially, a descriptive analysis was conducted on the sources, publications, and authors. Subsequently, bibliometric methodologies were applied, analyzing units such as authors, publications, journals, countries, and institutions to construct networks of knowledge, society, and collaboration (15). Specifically:

- Co-authorship Analysis: This technique explores the patterns of collaboration among individuals and organizations, aiding in identifying the social structures and collaborative networks at local, national, and international levels (16).
- Bibliographic Coupling: This is a measure of similarity that denotes the frequency with which two works cite a common third work. It can be inferred that the more references two works share, the more related their research topics are, which aids in identifying related prior research (17).
- Co-citation Analysis: Co-citation analysis is another metric to gauge disciplinary similarity, based on the frequency with which two works are cited by a third. A stronger relationship between the two works is indicated when they are frequently cited together by numerous other publications (18).

## 3 Results

The findings of this research are presented in the following section, covering various metrics: (a) scientific output, (b) source output, (c) author output, (d) production and collaboration networks across nations and institutions, and (e) keyword analysis.

### 3.1 Scientific output

**Figure 2:**
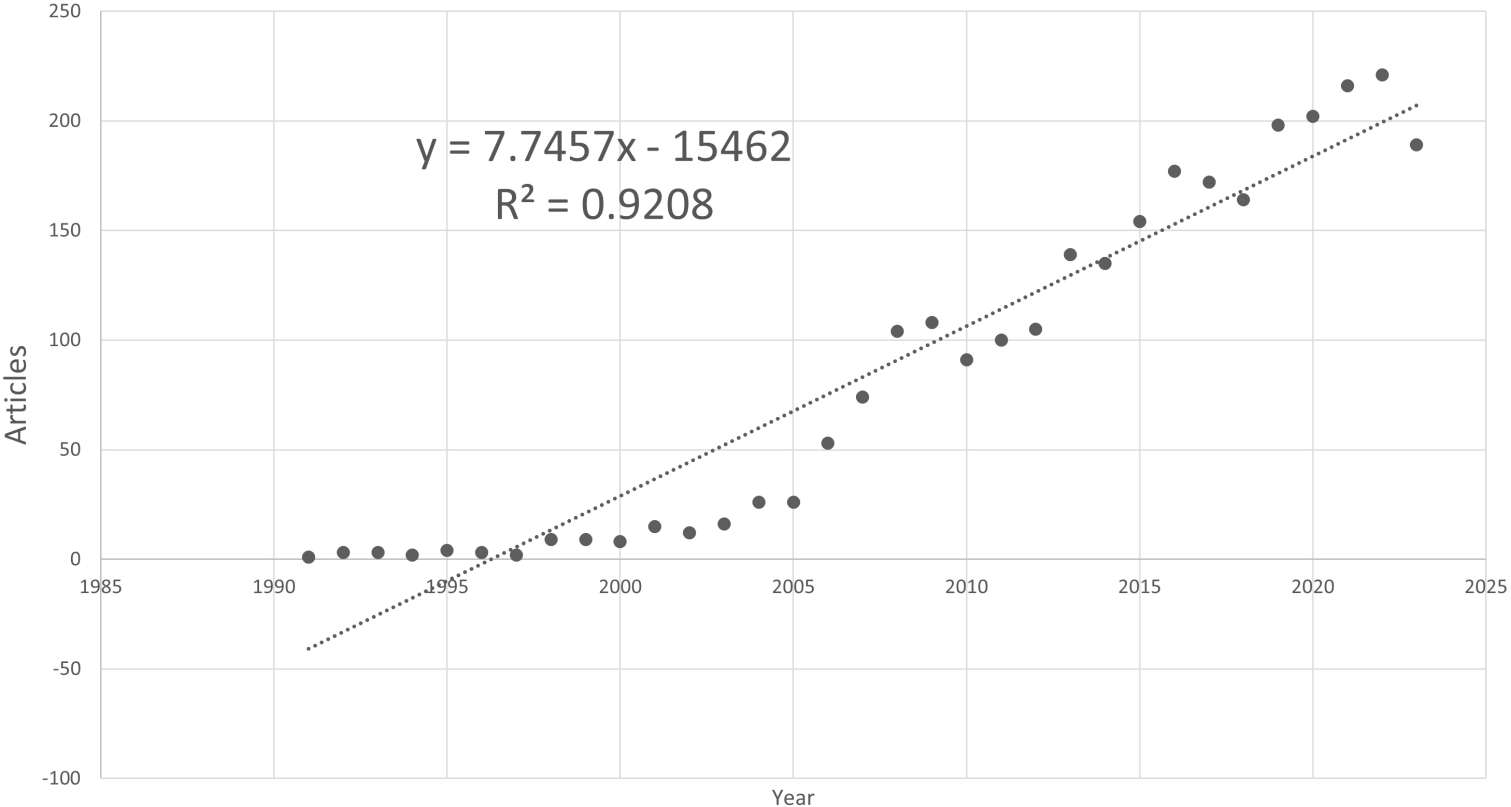
Annual publication quantities on prostate cancer and Inflammation from 2020 to 2024.

The study incorporated 2,210 articles (79.3%) and 576 reviews (20.7%), totaling 2,786 publications. **Figure 2** illustrates that the annual publication volume has consistently exceeded 150 over recent years. Although there was a slight declining trend in the last year, the trajectory still aligns with a regressive growth model, where R² = 0.9208. Publications from January 1, 2024, to April 12, 2024, were included for the current year but were not integrated into the regression analysis.

### 3.2 Sources of research

The number of journals that published papers related to prostate cancer and inflammation amounts to 857, encompassing both articles and reviews. **Table 1** displays sources that have published seven or more works on the topic, including three journals specifically dedicated to the field of prostate cancer research: “PROSTATE” (n = 18.3, 2%), “CANCERS” (n = 6.88%), and “PLOS ONE” (n = 5.72%). In **Table 1**, the term “article” represents the total number of research papers and reviews published by the journal, focusing on inflammation-related studies within the field of prostate cancer, as indexed in the Web of Science Core Collection (WOSCC) up until April 12, 2024.

**Table 1:** Main source productivity.

Sources	Articles
PROSTATE	157
CANCERS	59
PLOS ONE	49
INTERNATIONAL JOURNAL OF MOLECULAR SCIENCES	46
ONCOTARGET	43
CANCER EPIDEMIOLOGY BIOMARKERS & PREVENTION	41
JOURNAL OF UROLOGY	38
PROSTATE CANCER AND PROSTATIC DISEASES	37
UROLOGY	36
CANCER RESEARCH	35

**Figure 3:**
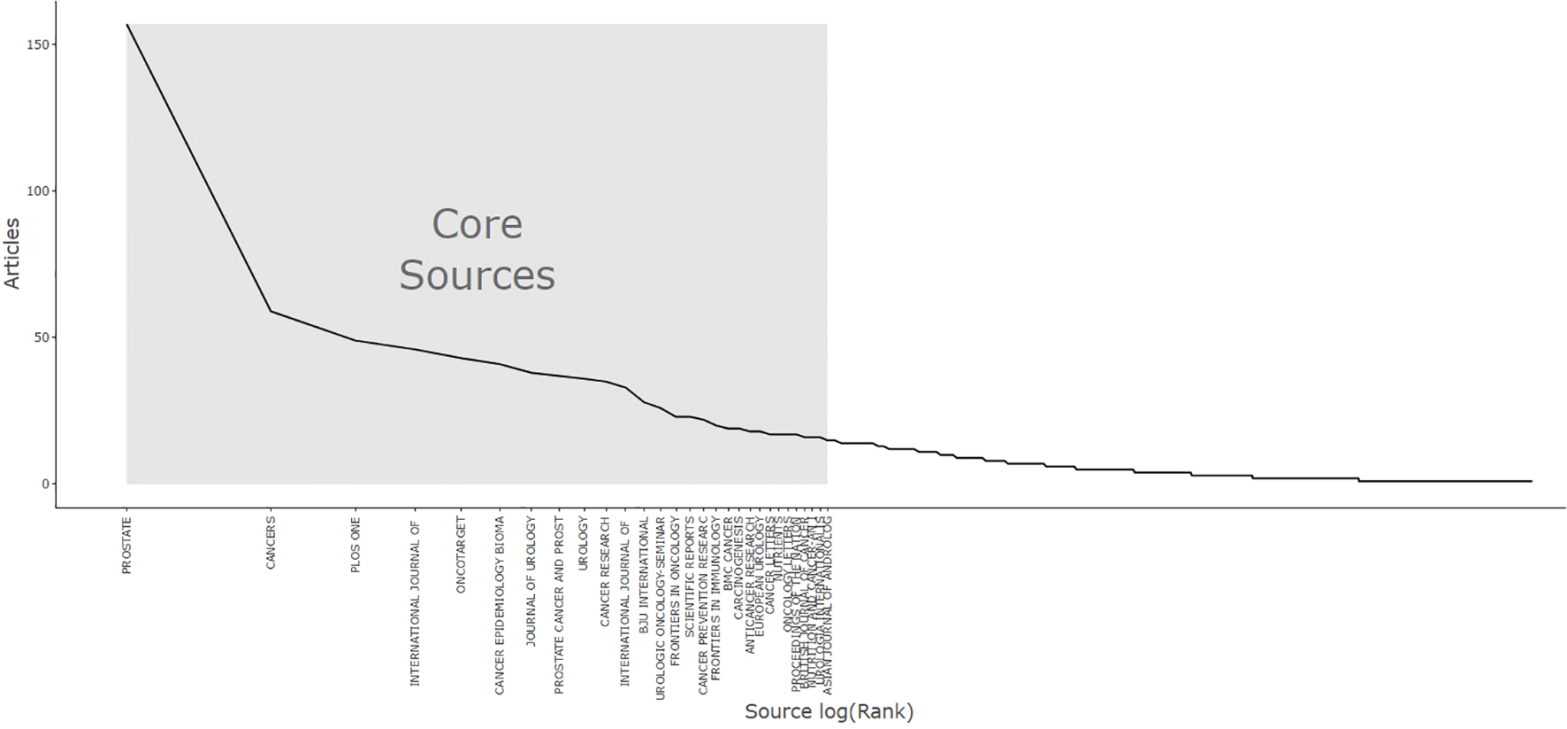
Bradford’s law core sources.

**Table 1** quantitatively supports Bradford’s Law for each source. According to this law, the distribution of scientific output related to a specific topic is highly skewed, with a minority of sources accounting for a majority of publications, while numerous sources contribute a comparably modest number of publications (19). **Figure 3** illustrates that the core sources for publications on prostate cancer and inflammation number 29, representing 3.38% of the total sample analyzed.

### 3.3 Authorial scientific production

This section delineates the publication output of authors, incorporating metrics such as co-authorship rates, collaboration indices, Lotka’s law estimates, and the publication volumes attributed to the most prolific countries/regions.

#### 3.3.1 Authors’ overall productivity

**Table 2** presents the principal descriptive variables pertaining to the number of authors who have published research on prostate cancer and inflammation. The total output related to this topic encompasses contributions from 14,053 authors, with an average of 19.82 publications per author. Only 79 authors (0.56%) have produced single-authored publications, whereas 25.02% of authors have engaged in multi-author endeavors, averaging 7.17 authors per publication.

**Table 2:** Authors main descriptive variables.

Variable	Results
Authors	14053
Authors of single-authored docs	77
Single-authored docs	79
Co-Authors per Doc	7.17
International co-authorships %	25.02%

**Figure 4:**
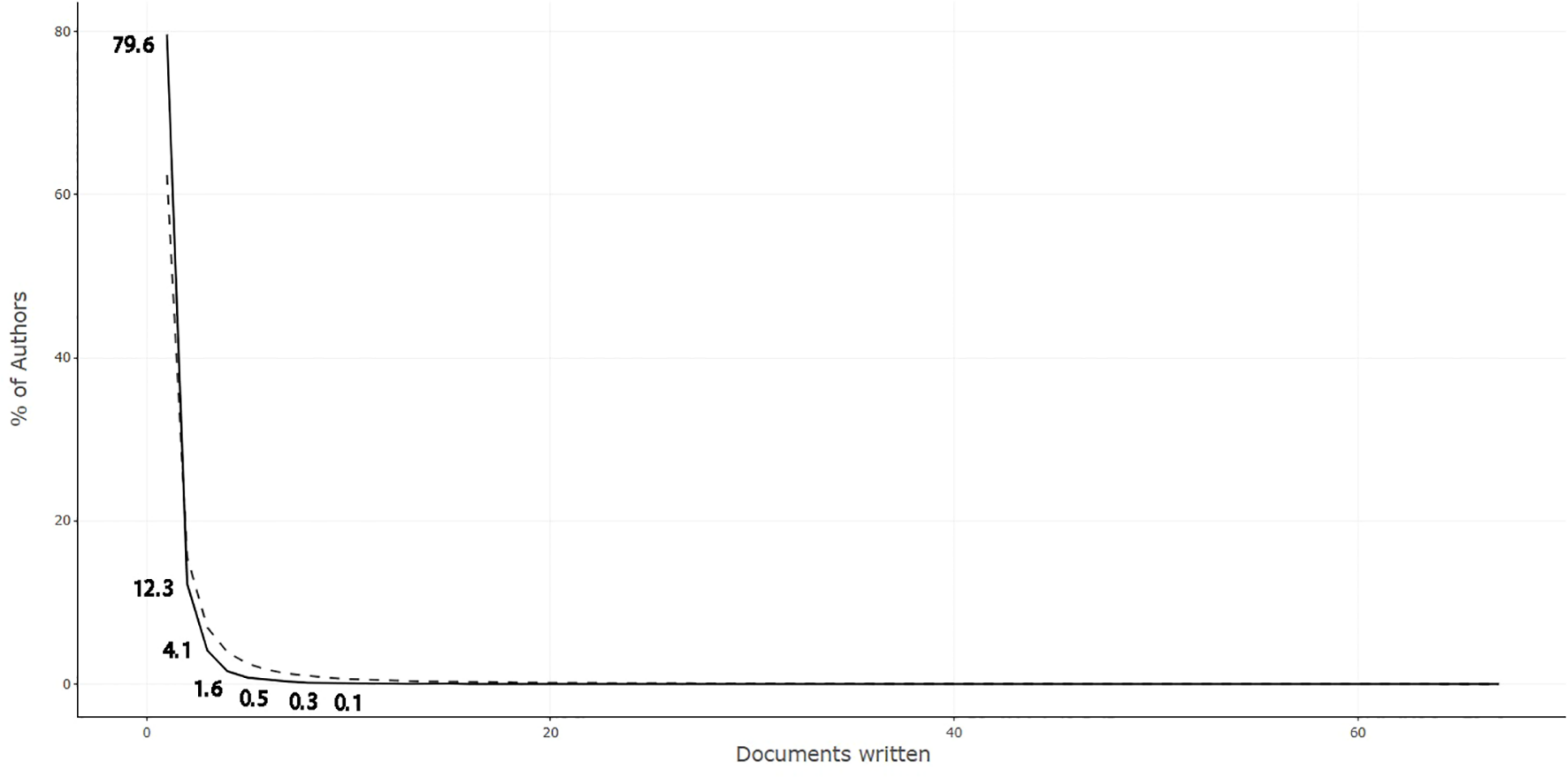
Lotka’s law coefficient estimation.

Similarly, utilizing Lotka’s coefficient estimates (17), the scientific output of authors is analyzed. The Lotka’s law distribution graph (**Figure 4**) presents a plot where the vertical axis denotes the proportion of authors for different quantities of literature relative to the total pool of authors, and the horizontal axis represents the number of documents. The dashed line in the graph represents a typical depiction of Lotka’s law. As indicated in **Table 3**, 11,188 authors have each published a single paper, accounting for 79.6% of the total, while 1,727 authors have published more than two papers, comprising 12.3% of the total. It is observable that the distribution of authors and the number of documents related to inflammation research in the prostate cancer domain closely follow the dashed line, generally conforming to the typical pattern of Lotka’s law. This principle suggests that the distribution of publication volumes among authors is inequitable, with few contributing significantly to the field, whereas the majority have authored only a single paper. The findings indicate that the vast majority of researchers in this domain related to inflammation in prostate cancer have only written one or two papers, suggesting that most scholars are relatively new to this area and the research is not yet deeply developed.

**Table 3:** Lotka’s law coefficient estimation distribution for authors.

Documents written	N. of Authors	Proportion of Authors
1	11188	0.796
2	1727	0.123
3	579	0.041
4	220	0.016
5	111	0.008
6	71	0.005
7	44	0.003
8	23	0.002
9	16	0.001
10	18	0.001

#### 3.3.2 Authorial publication volume

The publication volume of authors within this research domain is analyzed through various metrics including the number of publications, author dominance ranking, and co-citation analysis. **Table 4** delineates the publication volumes of different authors comprising the study sample within this field. These include the first author, the last author, and other positions in the authorship ranking. The authors with the highest publication counts are De Marzo Am and Platz E.A, with 67 and 44 publications respectively, followed by Isaacs W.B, Freedland S.J, Nelson W.G, and Drake C.G, among others. This analysis provides insights into the prolific contributors to the discourse on prostate cancer and inflammation.

**Table 4:** Number of articles by the top authors.

Authors	Articles	Articles Fractionalized
DE MARZO AM	67	9.63
PLATZ EA	44	5.07
ISAACS WB	33	4.04
FREEDLAND SJ	32	4.66
NELSON WG	30	5.99
DRAKE CG	27	2.42
THOMPSON IM	27	3.55
SFANOS KS	26	4.66
ANDRIOLE GL	21	2.98
MUCCI LA	19	1.69

### 3.4 Most cited publications

Co-citation refers to instances where a reference within one scholarly article is simultaneously cited by other publications. The frequency of citations serves as a vital indicator of a publication’s influence; the extent to which a document is cited reflects its value as a reference. The citation network formed through the inter-citing of published documents connects scholarly articles in a continuous system, thus ensuring the accumulation and transmission of knowledge (18). Analyzing co-citations allows for tracing the developmental trajectories within a field, swiftly identifying influential works, and making informed predictions about research trends and hotspots. Hence, in addition to the previously mentioned analyses, the study also examines the most influential documents, considering the number of citations they have garnered. **Table 5** lists the publications with the highest average annual citations (TC per year) in the Web of Science Core Collection database. Among these, the most cited documents are the works of Reuter S et al. and Calle EE et al., with citations numbering 3,547 and 2,611 respectively.

**Table 5:** Top 10 global cited works.

Paper DOI	Total Citations	TC per Year	Normalized TC
Reuter S, Gupta SC, Chaturvedi MM, Aggarwal BB. Oxidative stress, inflammation, and cancer: how are they linked? *Free Radic Biol Med*. 2010;49(11):1603-1616. doi:10.1016/j.freeradbiomed.2010.09.006	3547	236.47	34.94
Calle EE, Kaaks R. Overweight, obesity and cancer: epidemiological evidence and proposed mechanisms. *Nat Rev Cancer*. 2004;4(8):579-591. doi:10.1038/nrc1408	2611	124.33	15.00
Heinrich PC, Behrmann I, Haan S, Hermanns HM, Müller-Newen G, Schaper F. Principles of interleukin (IL)-6-type cytokine signalling and its regulation. *Biochem J*. 2003;374(Pt 1):1-20. doi:10.1042/BJ20030407	2453	111.50	11.43
Tilg H, Moschen AR. Adipocytokines: mediators linking adipose tissue, inflammation and immunity. *Nat Rev Immunol*. 2006;6(10):772-783. doi:10.1038/nri1937	2367	124.58	21.65
Hoesel B, Schmid JA. The complexity of NF-κB signaling in inflammation and cancer. *Mol Cancer*. 2013;12:86. Published 2013 Aug 2. doi:10.1186/1476-4598-12-86	2349	195.75	38.78
Taniguchi K, Karin M. NF-κB, inflammation, immunity and cancer: coming of age. *Nat Rev Immunol*. 2018;18(5):309-324. doi:10.1038/nri.2017.142	1617	231.00	39.32
DiDonato JA, Mercurio F, Karin M. NF-κB and the link between inflammation and cancer. *Immunol Rev*. 2012;246(1):379-400. doi:10.1111/j.1600-065X.2012.01099.x	1222	94.00	16.51
De Marzo AM, Platz EA, Sutcliffe S, et al. Inflammation in prostate carcinogenesis. *Nat Rev Cancer*. 2007;7(4):256-269. doi:10.1038/nrc2090	1207	67.06	14.08
Solinas G, Germano G, Mantovani A, Allavena P. Tumor-associated macrophages (TAM) as major players of the cancer-related inflammation. *J Leukoc Biol*. 2009;86(5):1065-1073. doi:10.1189/jlb.0609385	1095	68.44	15.79
Demierre MF, Higgins PD, Gruber SB, Hawk E, Lippman SM. Statins and cancer prevention. *Nat Rev Cancer*. 2005;5(12):930-942. doi:10.1038/nrc1751	657	32.85	5.41

Local citations represent those papers that have had a significant impact on this research field. Specifically, these are the publications within the analyzed literature collection that have received the highest number of citations. In this regard, the most frequently cited publications include the works of De Marzo AM et al. and S Fanos KS et al. (**Table 6**).

**Table 6:** Top 10 local citations.

Document DOI	Local Citations	Global Citations	LC/GC Ratio (%)	Normalized Local Citations	Normalized Global Citations
De Marzo AM, Platz EA, Sutcliffe S, et al. Inflammation in prostate carcinogenesis. *Nat Rev Cancer*. 2007;7(4):256-269. doi:10.1038/nrc2090	518	1207	42.92	38.33	14.08
Sfanos KS, De Marzo AM. Prostate cancer and inflammation: the evidence. *Histopathology*. 2012;60(1):199-215. doi:10.1111/j.1365-2559.2011.04033.x	247	439	56.26	39.54	5.93
Gurel B, Lucia MS, Thompson IM Jr, et al. Chronic inflammation in benign prostate tissue is associated with high-grade prostate cancer in the placebo arm of the prostate cancer prevention trial. *Cancer Epidemiol Biomarkers Prev*. 2014;23(5):847-856. doi:10.1158/1055-9965.EPI-13-1126	122	174	70.11	23.13	3.47
Palapattu GS, Sutcliffe S, Bastian PJ, et al. Prostate carcinogenesis and inflammation: emerging insights. *Carcinogenesis*. 2005;26(7):1170-1181. doi:10.1093/carcin/bgh317	109	281	38.79	6.50	2.31
De Nunzio C, Kramer G, Marberger M, et al. The controversial relationship between benign prostatic hyperplasia and prostate cancer: the role of inflammation. *Eur Urol*. 2011;60(1):106-117. doi:10.1016/j.eururo.2011.03.055	105	328	32.01	19.81	5.92
Sfanos KS, Yegnasubramanian S, Nelson WG, De Marzo AM. The inflammatory microenvironment and microbiome in prostate cancer development. *Nat Rev Urol*. 2018;15(1):11-24. doi:10.1038/nrurol.2017.167	95	262	36.26	27.87	6.37
Nguyen DP, Li J, Tewari AK. Inflammation and prostate cancer: the role of interleukin 6 (IL-6). *BJU Int*. 2014;113(6):986-992. doi:10.1111/bju.12452	83	266	31.20	15.74	5.31
Ammirante M, Luo JL, Grivennikov S, Nedospasov S, Karin M. B-cell-derived lymphotoxin promotes castration-resistant prostate cancer. *Nature*. 2010;464(7286):302-305. doi:10.1038/nature08782	66	466	14.16	12.54	4.59
Cohen RJ, Shannon BA, McNeal JE, Shannon T, Garrett KL. Propionibacterium acnes associated with inflammation in radical prostatectomy specimens: a possible link to cancer evolution?. *J Urol*. 2005;173(6):1969-1974. doi:10.1097/01.ju.0000158161.15277.78	64	173	36.99	3.82	1.42
Karazanashvili G. Editorial comment on: the relationship between prostate inflammation and lower urinary tract symptoms: examination of baseline data from the REDUCE trial. *Eur Urol*. 2008;54(6):1383-1384. doi:10.1016/j.eururo.2007.11.027	64	334	19.16	9.31	5.15

### 3.5 National and institutional publication volumes

The purpose of this segment of analysis is to examine the publication volumes from various nations and institutions and to establish their networks of collaboration.

#### 3.5.1 National publication volume

In terms of contributions to the topic by country, three nations have each published more than 200 articles. Specifically, the United States has produced 954 publications; China has contributed 435 publications; and Italy has issued 215 publications (**Table 7**).

**Table 7:** Top 10 country production and corresponding authors’ country.

Country	Articles	SCP	MCP	Freq	MCP Ratio
USA	954	746	208	0.342	0.218
CHINA	435	373	62	0.156	0.143
ITALY	215	147	68	0.077	0.316
JAPAN	104	87	17	0.037	0.163
UNITED KINGDOM	84	48	36	0.03	0.429
KOREA	80	65	15	0.029	0.188
GERMANY	70	47	23	0.025	0.329
TURKEY	63	60	3	0.023	0.048
AUSTRALIA	54	34	20	0.019	0.37
CANADA	54	31	23	0.019	0.426

*SCP, Single Country Publications; MCP, Multiple Country Publications.

Regarding the nationality of corresponding authors, the study indicates that international collaboration among countries is notably scant. Overall, there is a relative lack of scientific cooperation among nations, with substantial room for enhancement in collaborative outputs. Only the United Kingdom exhibits the highest rate of collaboration, with a ratio of 0.42. This suggests that out of seven publications, approximately three involve authors from different countries.

#### 3.5.2 Institutional publication volume

As illustrated in **Table 8**, within the domain of prostate inflammation, the institution contributing the most publications is Johns Hopkins University, followed by the University of California system.

**Table 8:** Top 10 institutions by publication volume.

Affiliation	Articles
JOHNS HOPKINS UNIVERSITY	334
UNIVERSITY OF CALIFORNIA SYSTEM	233
HARVARD UNIVERSITY	226
JOHNS HOPKINS MEDICINE	154
UNIVERSITY OF TEXAS SYSTEM	151
UNIVERSITY OF CALIFORNIA LOS ANGELES	121
NIH NATIONAL CANCER INSTITUTE (NCI)	120
NATIONAL INSTITUTES OF HEALTH (NIH) - USA	114
HARVARD MEDICAL SCHOOL	93
UNIVERSITY SYSTEM OF OHIO	89

#### 3.5.3 Institutional and national collaboration networks

**Figure 5:**
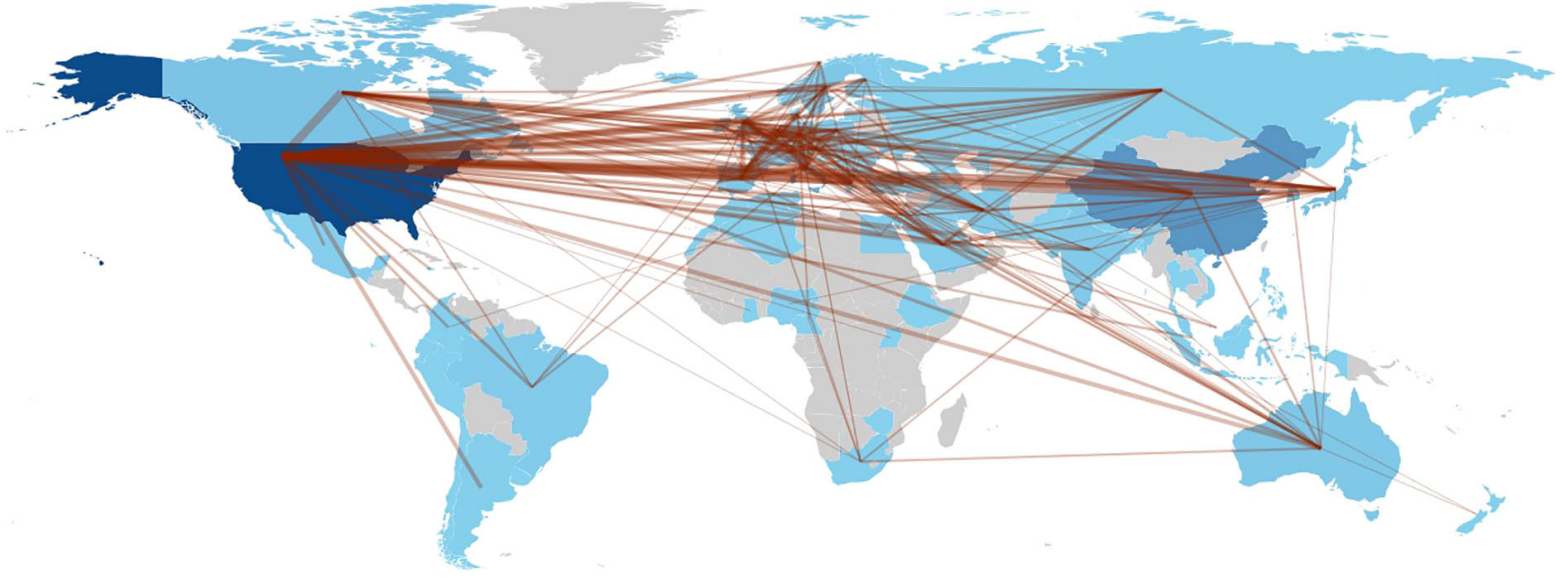
Country collaboration map.

The network of institutional and national collaborations provides an intricate overview of the interactions among nations and organizations, illustrating the depth of academic exchange as reflected by the granularity of these networks. The volume of publications per country signifies the investment in research within specific scholarly domains. As depicted in **Figure 5**, the United States, the United Kingdom, Germany, and Italy emerge as prominent partners in research relevant to the study’s themes. Specifically, the United States predominantly collaborates with European nations (the United Kingdom and Italy) and Asian countries (China and Japan), as well as Canada. Meanwhile, the United Kingdom engages primarily with the United States, Switzerland, Italy, Greece (Athens), and France. Germany’s collaborations are chiefly with the United States and Italy, whereas Italy frequently partners with the United States, the United Kingdom, and Spain. Lastly, China’s main collaborations are with the United States, the United Kingdom, Canada, and Greece (Athens).

### 3.6 Keyword analysis

The recurrent high-frequency keywords significantly mirror the foundational research, emerging hotspots, and evolving trends within the field. **Table 9** enumerates the predominant high-frequency keywords currently relevant in prostate-related inflammation research. Beyond the core keywords central to this study—such as “prostate cancer,” “inflammation,” “cancer,” and “prostatic neoplasms”—the table also highlights additional significant terms including “prostate-specific antigen,” “apoptosis,” “cytokines,” “oxidative stress,” “tumor microenvironment,” and “biomarker.” These terms collectively delineate the focal areas of scholarly attention and inquiry in this domain.

**Table 9:** Frequency of author keywords.

Terms	Frequency	Terms	Frequency
prostate cancer	1127	androgen receptor	29
inflammation	612	biomarkers	29
cancer	198	chronic inflammation	26
prostate	129	cox-2	26
prostatitis	86	metabolic syndrome	26
prostatic neoplasms	85	castration-resistant prostate cancer	25
prostate-specific antigen	76	diet	24
apoptosis	73	immunotherapy	24
benign prostatic hyperplasia	69	interleukin-6	24
cytokines	61	invasion	24
oxidative stress	61	microbiome	24
tumor microenvironment	55	radical prostatectomy	24
prognosis	53	radiotherapy	24
metastasis	51	epidemiology	23
obesity	47	stat3	23
angiogenesis	45	macrophages	22
nf-kappa b	45	neutrophil-to-lymphocyte ratio	22
prostate biopsy	42	proliferation	22
biomarker	41	chemoprevention	21
biopsy	38	immunohistochemistry	21
meta-analysis	38	infection	21
c-reactive protein	33	breast cancer	20
psa	32	macrophage	20
il-6	31	androgen deprivation therapy	19
polymorphism	30	prostatic hyperplasia	19

**Figure 6:**
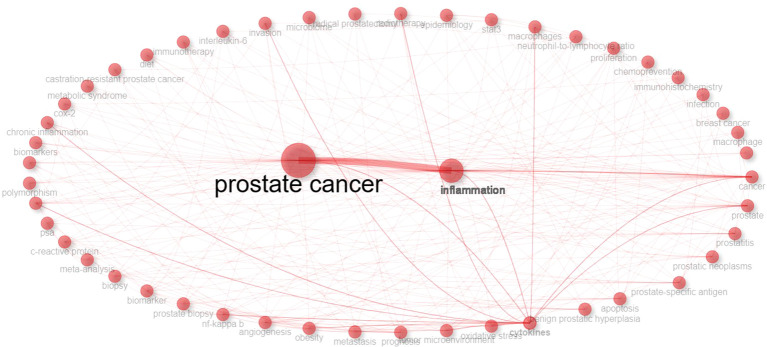
Co-occurrence network of author keywords.

This study further explores the co-occurrence network of keywords, which serves as a high-level abstraction of the content and themes of academic papers, directly reflecting the current research hotspots and enhancing understanding of trends in inflammation-related studies within the domain of prostate cancer. The keyword co-occurrence network elucidates the connections between literature keywords, thus deepening our comprehension of the knowledge structure in this field. In **Figure 6**, each node represents a keyword, with larger nodes indicating a higher frequency of occurrence. The relationships among these keywords reveal close links between research themes, with high-frequency keywords marking the hotspots of the field (19).

Surrounding the primary keywords of “prostate cancer” and the core topic of “inflammation,” additional keywords emerge, highlighting central themes across various research areas. Analyzing the centrality and categorization of these keywords in the diagram, it is evident that the research hotspots related to inflammation predominantly focus on areas including “prostate-specific antigen,” “cytokines,” “oxidative stress,” “tumor microenvironment,” “prognosis,” and “obesity,” all of which are intricately linked to the topics of inflammation and prostate cancer.

**Figure 7:**
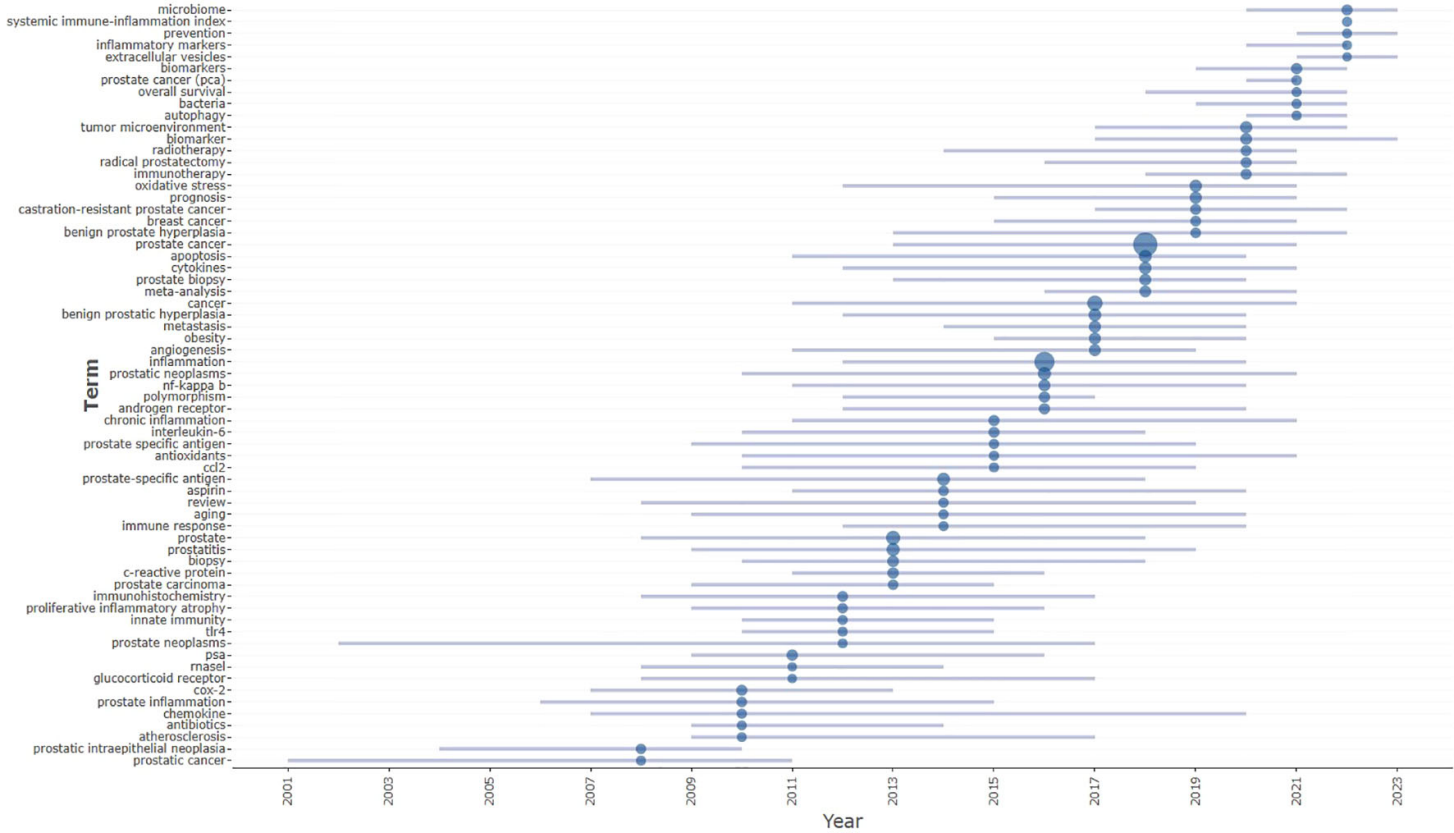
Trend graph of keywords.

In **Figure 7**, the horizontal bars and dots beside each keyword indicate the frequency of occurrence of these terms in the literature across different years. Longer bars and more dots beneath a keyword signify its extensive use and discussion over a specific period. For instance, keywords such as “prostate cancer (PCa),” “biomarker,” and “inflammation” are shown to have a wide temporal span and high frequency, indicating their significant and sustained relevance in the research field.

By tracking changes in keyword prevalence over time, shifts in research focus become apparent. For example, if terms like “angiogenesis” were prevalent in earlier years, but more recent years have seen an increase in terms like “microbiome,” “systemic immune-inflammation index,” and “inflammatory markers,” it suggests a shift in research interest from traditional angiogenesis to studies focused on the microbiome, inflammation indices, and inflammatory markers. The occurrence of these keywords helps trace the historical trajectory and evolution of academic discourse. This data constructs a historical narrative of a research topic and its evolution over time.

The strategic map, based on density and centrality, delineates the relative positioning of concepts within the map, thereby revealing the dynamics and importance of various themes within a specific academic field. This graphical representation is commonly used to exhibit the developmental trends and centrality of different themes within a particular research area. Hence, concepts positioned at the center of the image in the conceptual map possess greater transcendence, while those deviating from the center demonstrate lesser transcendence. The vertical axis, labeled “Development (or Density),” indicates the concentration and maturity of research within a specific area. The horizontal axis, “Relevance (or Centrality),” denotes the centrality and influence of themes across the entire research field. Generally:

- Motor Themes in the top right quadrant (Quadrant I), which are significant and well-developed foundational themes;
- Niche Themes in the top left quadrant, representing highly developed themes that are less critical to the current field;
- Emerging or Declining Themes in the bottom left quadrant, which are either nascent or diminishing themes;
- Basic Themes in the bottom right quadrant, encompassing general, transversal, and foundational themes that are not well-developed.

**Figure 8:**
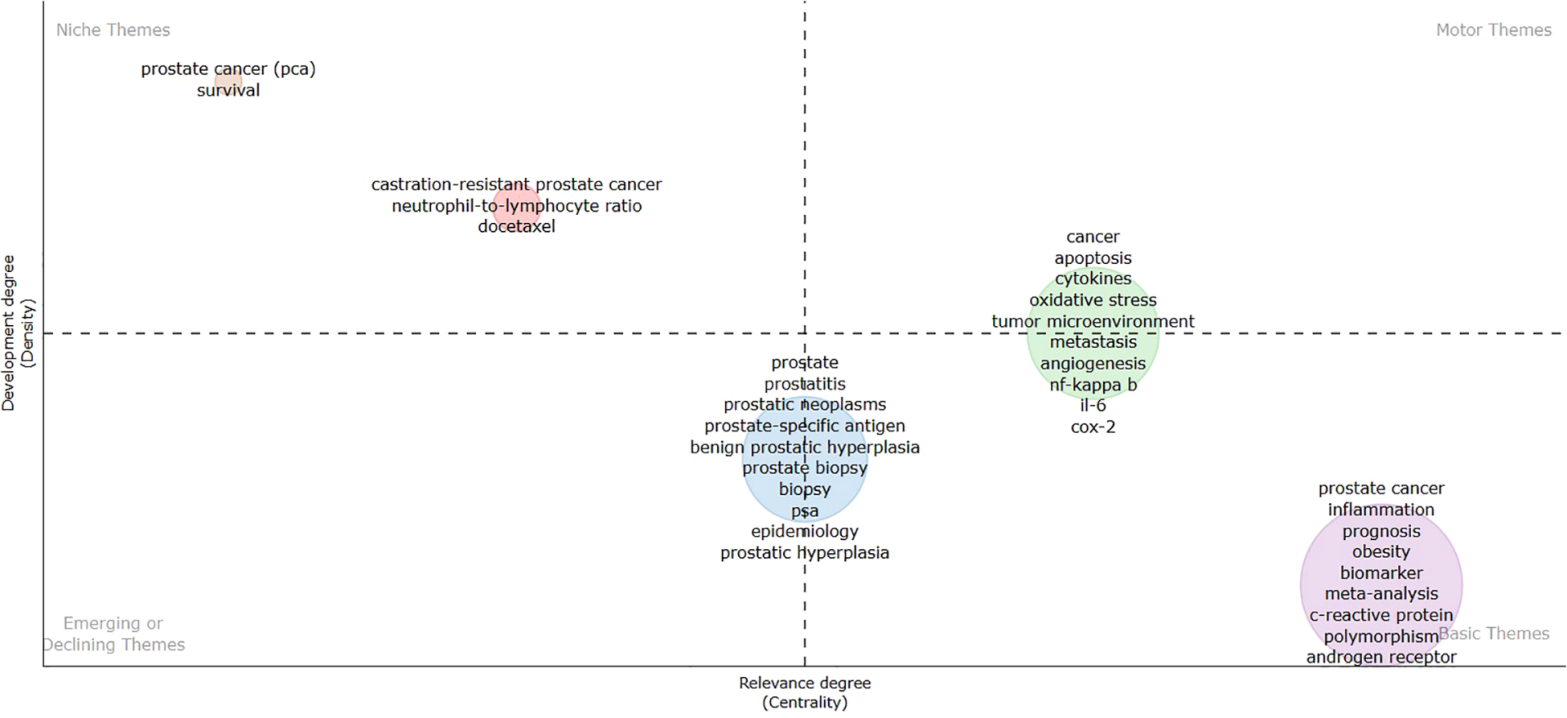
Strategic map for inflammation-related research in prostate cancer.

**Figure 8** displays the strategic map for inflammation-related research in the field of prostate cancer. Niche Themes (top left), such as “prostate cancer (PCa) survival” and “castration-resistant prostate cancer,” have been extensively researched but have a relatively minor impact on the broader field of prostate cancer research. Motor Themes (top right), like “cancer,” “apoptosis,” and “cytokines,” are central issues within prostate cancer research, receiving broad attention and extensive study. Emerging or Declining Themes (bottom left) are not significantly marked on this map, possibly due to the selected research focusing on more mature topics. Basic Themes (bottom right), such as “prostate cancer,” “inflammation,” “prognosis,” “obesity,” and “biomarker,” represent foundational issues in prostate cancer research that continue to receive sustained attention. Themes like “cancer” and “apoptosis” might be the focal points of most studies, reflecting the activity and importance of these areas. Specific therapeutic approaches and clinically relevant themes, like “castration-resistant prostate cancer” and “docetaxel,” are well-researched but may not currently be the main drivers of research.

**Figure 9:**
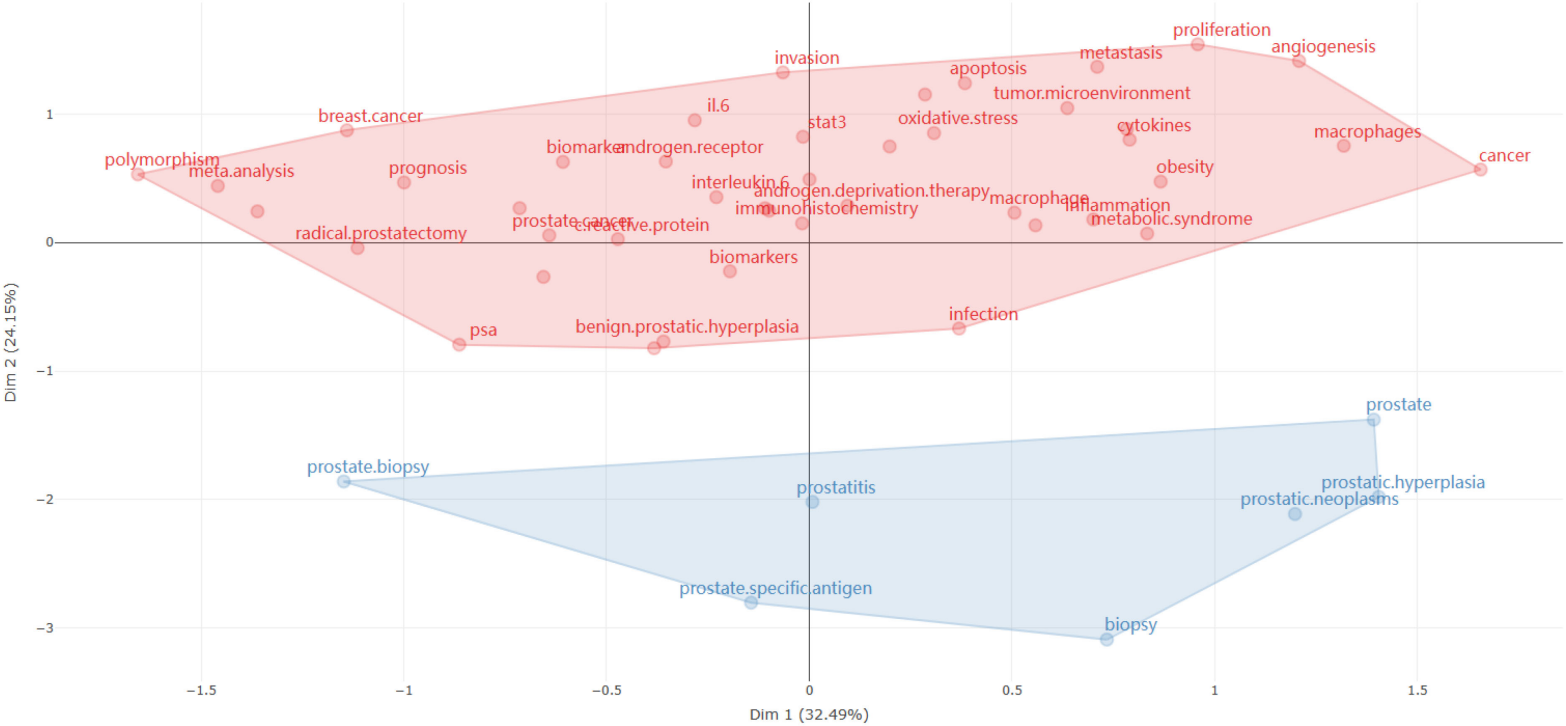
Cluster analysis of author keywords using multiple correspondence analysis.

The cluster analysis of high-frequency keywords is conducted using multiple correspondence analysis, resulting in a conceptual structure map of authors’ keywords, which is depicted in a two-dimensional plot (**Figure 9**). The proximity of points representing each keyword on the map indicates a similarity in their distribution, suggesting they appear more frequently together within the literature. Moreover, the closeness of a keyword to the central point on the map signifies its popularity within the research field. Keywords situated near the center receive substantial attention from the research community, whereas those on the periphery have lower relevance to other dominant research themes. Observations from **Figure 9** include:

1. Red Area: Represents keywords associated with oncology, particularly focusing on prostate and breast cancer, encompassing biomarkers, therapeutic approaches, and prognostic factors. Terms such as “breast cancer,” “biomarkers,” “androgen receptor,” “invasion,” and “metastasis” are directly linked to tumor growth, spread, and treatment.
2. Blue Area: Concentrates on keywords related to prostate disease, typically associated with non-cancerous conditions like “prostatitis” (prostate inflammation) and “prostatic hyperplasia” (prostate enlargement).

Keywords such as “polymorphism” and “meta-analysis” are usually associated with statistical methods and genetic research, positioned in the upper left of the map, indicating these terms may stand relatively independent from other medical terms. Conversely, “inflammation,” “oxidative stress,” and “cytokines” are located in the upper right, likely suggesting strong connections with immunology and biochemistry.

## 4 Discussion

**Figure 10:**
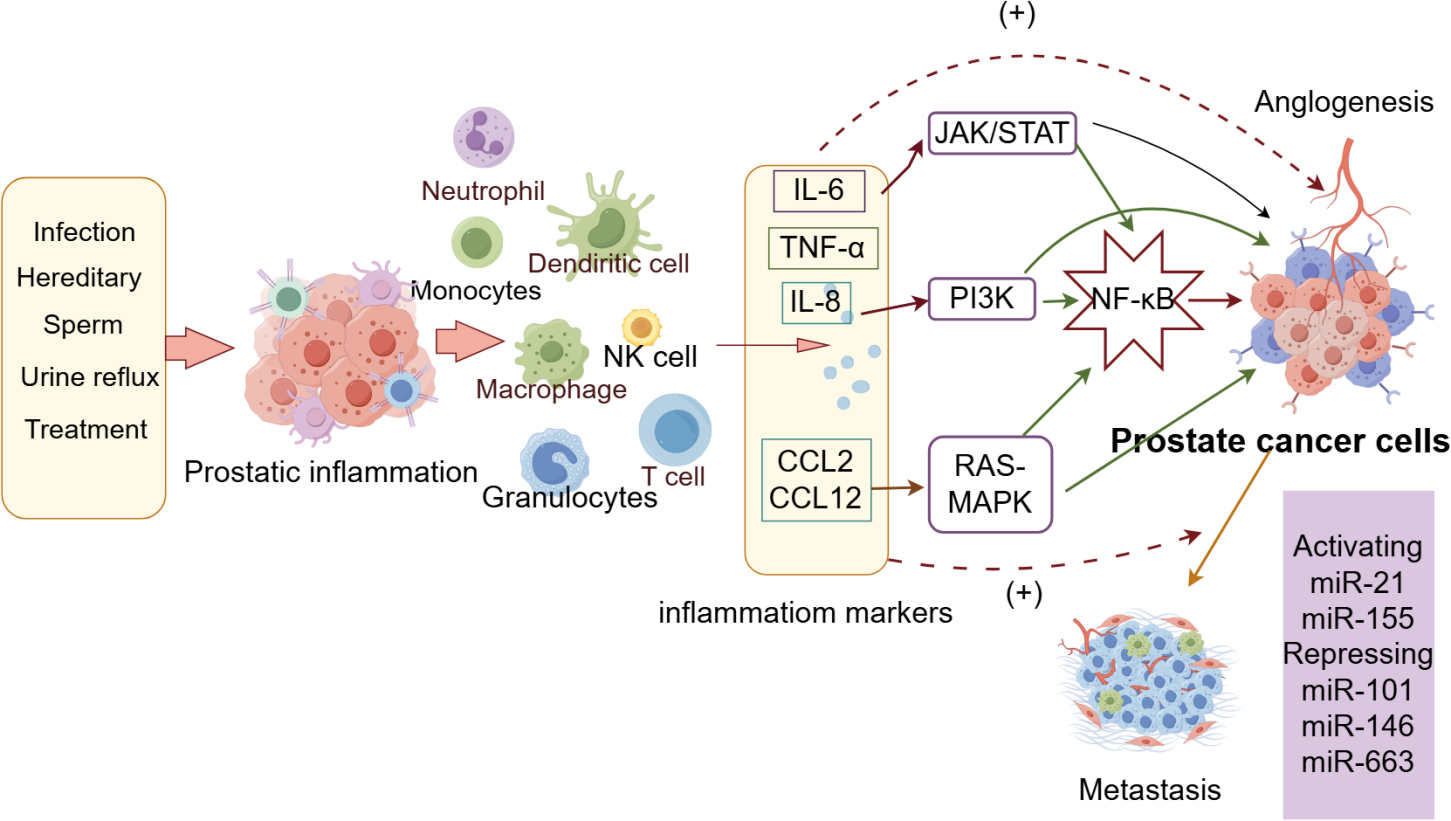
Simplified schematic of the processes involved in the progression of prostate cancer induced by inflammation.

Recent interest has surged in the pro-tumorigenic role of the inflammatory microenvironment, largely driven by a robust body of clinical, molecular, histopathological, and epidemiological evidence linking prostate cancer with concurrent inflammatory complications. Multiple factors, such as infections, genetics, seminal and urinary reflux, and treatments, can incite prostate inflammation. The inflammatory response engages a variety of immune cells, including neutrophils, dendritic cells, natural killer (NK) cells, macrophages, T cells, and granulocytes. Cytokines produced during this process, such as interleukin-6 (IL-6), tumor necrosis factor-alpha (TNF-α), and interleukin-8 (IL-8), along with chemokines like CCL2 and CCL12, are thought to be pivotal in bridging the progression to prostate cancer. These factors activate signaling pathways including JAK/STAT, PI3K, and RAS-MAPK, which in turn trigger the activation of nuclear factor kappa-light-chain-enhancer of activated B cells (NF-κB), a crucial transcription factor that regulates a myriad of cellular functions, including cell survival, proliferation, and apoptosis. In prostate cancer cells, NF-κB promotes angiogenesis and tumor metastasis. MicroRNAs (miRNAs), which can either promote or inhibit tumor development through the regulation of gene expression, also play a significant role in this context. Active miRNAs such as miR-21 and miR-155, along with suppressive miRNAs like miR-101, miR-146, and miR-663, are instrumental in this process (20), as illustrated in **Figure 10**.

Inflammation is a significant driver of prostate cancer risk and profoundly impacts the tumor microenvironment, facilitating the progression to advanced, treatment-resistant disease. Although acute inflammatory responses are crucial for clearing pathogenic infections, prolonged and uncontrolled inflammation can result in cellular and tissue damage. Chronic inflammation is associated with an increased risk of malignancies such as prostate cancer (11). As illustrated in **Table 9**, keywords like microbiota, radiotherapy, gene expression, prostatectomy, IL-6, IL-1β, TNF-α, and STAT3 hold central positions within the prostate cancer domain, reflecting their frequent study. Research indicates that chronic inflammation within the prostate microenvironment can alter the tumor milieu, promoting cancer progression through mechanisms such as proliferation, cell survival, immune evasion, tissue remodeling, angiogenesis, metastasis, and treatment resistance (21). When cells are damaged or infected, they release agents that activate inflammatory signaling pathways, releasing inflammatory mediators and cytokines, recruiting inflammatory immune cells, and increasing vascular permeability (22). NF-κB, a transcription factor primarily activated by cytokines such as tumor necrosis factor-alpha (TNF-α), plays a pivotal role in this inflammatory signaling pathway. Once activated, this transcription factor regulates the expression of cytokines and factors involved in cancer development and progression, such as IL-6 for tumor cell survival, the angiogenic factor VEGF, and IL-8, further producing mediators for immune cell recruitment. IL-6, a marker of chronic inflammation, is well-studied in cancer (23). Elevated serum levels of IL-6 in metastatic and castration-resistant prostate cancer (CRPC) patients correlate significantly with tumor staging and negatively with survival and treatment response (24). TNF-α is a key inflammatory mediator; elevated serum TNF-α is found in 76% of patients with recurrent disease, and those with high levels face a higher mortality rate (25). A retrospective study analyzed a range of inflammatory cytokines and chemokines concerning their relationship with prostate cancer and progression to advanced disease. It was determined that elevated serum levels of IL-8, TNF-α, and CCL2 are associated with accelerated progression to castration-resistant disease and correlate with poorer overall survival in prostate cancer patients (26). Therefore, in recent years, the link between inflammation and prostate cancer has garnered significant interest. Unfortunately, despite notable progress, the predictive value and prognostic research of inflammation-related markers in prostate cancer still have limitations (27). Recently, bibliometric analysis has been extensively utilized to clarify the current state of research fields and track trends (28). This study represents the first systematic literature search to create a knowledge map and forecast future research on inflammation-related studies in prostate cancer. Keywords such as meta-analysis, systemic immune-inflammation index, inflammation markers, and tumor microenvironment are significant in this field but are underdeveloped, with sparse literature, necessitating further research by scientists.

In 2020, De Bono JS and colleagues highlighted that intra-prostatic inflammation is a risk factor for the development of prostate cancer, associated with dietary habits, chemical injuries, and altered microbiota. The recruitment and amplification of inflammatory cells within the prostate can promote DNA double-strand breaks and activation of androgen receptors in prostate epithelial cells. The activation of an aging-related secretory phenotype can further trigger an “inflammatory storm,” leading to additional DNA damage. This stimulates the overexpression of DNA repair and tumor suppressor genes, making these genes more susceptible to mutagenic factors, while defects in germline DNA repair genes accelerate cancer onset (29).

In 2021, Matsushita M and others conducted a study with 152 Japanese men undergoing prostate biopsies to elucidate the association between human gut microbiota and prostate cancer (PCa). Their analysis identified specific bacterial groups associated with high-risk PCa, suggesting that the gut microbiome profile could be a novel and useful biomarker for detecting high-risk PCa and may even have a carcinogenic role in PCa (30).

In 2023, Şahin E and colleagues explored the prognostic role of the neutrophil-to-lymphocyte ratio (NLR), platelet-to-lymphocyte ratio (PLR), systemic immune-inflammation index (SII), and systemic immune-inflammation value (PIV) in patients undergoing treatment with 177Lu–PSMA-617 for metastatic castration-resistant prostate cancer. Their findings indicated that NLR, PLR, SII, and PIV are associated with prognosis in these patients. Higher values of NLR, PLR, SII, and PIV are linked to poorer prognostic indicators, such as shorter overall survival and progression-free survival, suggesting that these markers could serve as valuable biomarkers for predicting the prognosis of patients with metastatic castration-resistant prostate cancer (31).

Bouras E and colleagues employed the Mendelian randomization (MR) approach to investigate the relationship between chronic inflammation and the risk of various cancers. They used genotypes as instrumental variables to represent the concentrations of cancer risk-related circulating cytokines, assessing the causal relationships between sustained cytokine levels and multiple cancers. Their results indicated that high plasma concentrations of specific cytokines are positively associated with the risk of prostate cancer (32).

Inflammation plays a pivotal role in the initiation and progression of prostate cancer. Cytokines and chemokines released by inflammatory cells such as macrophages and T cells can induce oxidative stress, leading to DNA damage and an increased risk of mutations that drive cancer development and progression. This has sparked significant interest among researchers in targeting key enzymes and signaling pathways involved in the inflammatory response—such as COX-2, NF-κB, IL-6, and IL-1β—as potential therapeutic strategies for prostate cancer. Current therapies, including COX-2 inhibitors and NF-κB pathway inhibitors, show promise but are hindered by challenges such as poor target specificity, resistance, and patient heterogeneity. Additionally, the tumor microenvironment, influenced by chronic inflammation, may contribute to treatment resistance and tumor progression, further complicating therapeutic strategies. Understanding the interplay between inflammatory pathways and other oncogenic processes is crucial for designing more effective therapies. To overcome these obstacles, future research should prioritize the identification of reliable biomarkers, the development of combination therapies that simultaneously target multiple pathways, and the execution of large-scale clinical trials to assess the feasibility and efficacy of inflammation-targeted therapies in prostate cancer (33, 34). Moreover, exploring the potential of combining immunotherapy with inflammation-targeted treatments could open new avenues for enhancing immune responses against prostate cancer while mitigating the effects of chronic inflammation.

This study represents the first to describe and visualize the knowledge landscape of inflammation-related research within the domain of prostate cancer. Compared to previous reviews and meta-analyses, this research offers distinct advantages. We utilized bibliometric tools to visualize data, enhancing the specificity and richness of the results. Like other studies, this one has its limitations. Firstly, our data originates from the Web of Science (WoS) database, necessitating the inclusion of additional relevant literature. Secondly, the language of the publications included in this study is limited to English, which may lead to the omission of significant works in other languages. Lastly, the absence of standardized criteria for manual selection could introduce selection bias in data retrieval.

Through bibliometric analysis, this study elucidates the contributions of countries, institutions, authors, and journals to the field of inflammation and prostate cancer. It identifies research hotspots and frontiers within the area. Notably, topics such as “inflammation-related biomarkers,” “biomarkers,” and “tumor microenvironment” are emerging as focal points and frontiers in the fields of inflammation and prostate cancer. These findings provide researchers with a clearer understanding of the relationship between inflammation and prostate cancer, underscoring the need for timely updates and ongoing tracking of developments in this crucial area of study.

## 5 Conclusion

This marks the inaugural foray into the realm of bibliometrics to ascertain the status quo of inflammation-related research within the domain of prostate cancer, alongside endeavors to prognosticate certain forthcoming trends. Building upon extant scholarship, it further enriches the discourse on inflammation-mediated cancer therapeutics. Noteworthy is the substantial contribution of both the United States and China to this sphere. Analysis delineates “inflammation-related biomarkers,” “inflammation indices,” and the “tumor immune microenvironment” as focal points and frontiers within this domain. With burgeoning exploration into tumor immunology, the potential of inflammation-related biomarkers in advancing the diagnostic and therapeutic frontiers of prostate cancer looms large. They hold promise as pivotal domains for future inquiry and bear significant relevance for clinical application in the diagnosis and treatment of prostate cancer.

## Data Availability

The original contributions presented in the study are included in the article/supplementary material. Further inquiries can be directed to the corresponding author.
